# Berberine represses Wnt/β-catenin pathway activation via modulating the microRNA-103a-3p/Bromodomain-containing protein 4 axis, thereby refraining pyroptosis and reducing the intestinal mucosal barrier defect induced via colitis

**DOI:** 10.1080/21655979.2022.2047405

**Published:** 2022-03-08

**Authors:** Xun Zhao, DeJun Cui, WenQiang Yuan, Chen Chen, Qi Liu

**Affiliations:** a The Graduate School, Guizhou Medical University, Guiyang City, Guizhou Province, China; b Department of Gastroenterology, Guizhou Provincial People’s Hospital, Guiyang City, Guizhou Province, China

**Keywords:** Colitis, Berberine, microRNA-103a-3p, Bromodomain-containing protein 4, intestinal mucosal barrier defect

## Abstract

Intestinal barrier dysfunction is inflammatory bowel disease’s hallmark. Berberine (BBR) has manifested its anti-inflammatory properties in colitis. For exploring the molecular mechanism of BBR’s impacts on colitis, application of a dextran sodium sulfate-induced mouse colitis *in vivo* model was with recording the body weight, stool consistency, stool occult blood and general physical symptoms of all groups of mice every day. Behind assessment of intestinal permeability, detection of colon damage’s degree and apoptosis, and inflammatory factors for assessment of pyroptosis was conducted. Application of interleukin-6-stimulated Caco-2 cells was for construction of an *in vitro* model. Then detection of cell advancement with inflammation and measurement of the barrier’s integrity were put into effect. Verification of microRNA (miR)-103a-3p and Bromodomain-containing protein 4 (BRD4)’s targeting link was conducted. Experiments have clarified BBR, elevated miR-103a-3p or repressive BRD4 was available to alleviate colitis-stimulated pyroptosis and intestinal mucosal barrier defects. BBR elevated miR-103a-3p to target BRD4; Refraining miR-103a-3p or enhancive BRD4 turned around BBR’s therapeutic action on colitis injury. BBR depressed Wnt/β-catenin pathway activation via controlling the miR-103a-3p/BRD4 axis. All in all, BBR represses Wnt/β-catenin pathway activation via modulating the miR-103a-3p/BRD4 axis, thereby mitigating colitis-stimulated pyroptosis and the intestinal mucosal barrier defect. The research suggests BBR is supposed to take on potential in colitis cure.

## Graphical Abstract

**Figure:**
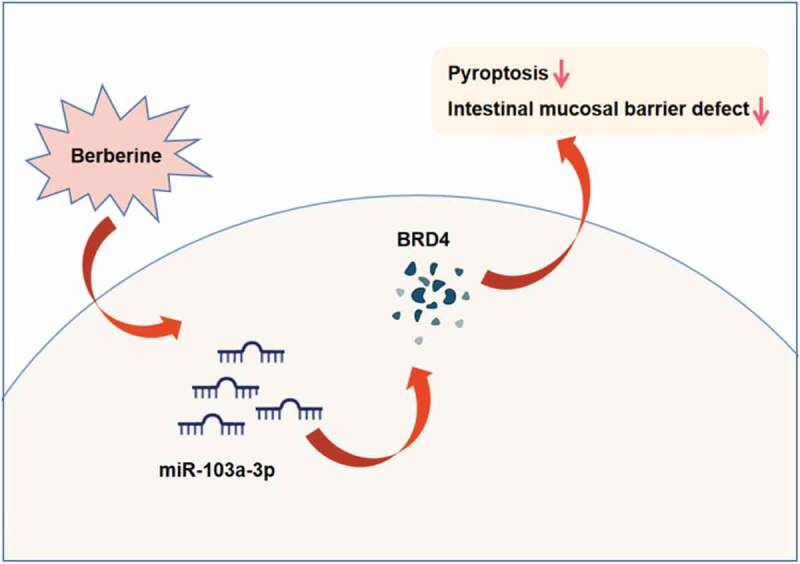

## 1. Introduction

The intestinal microbiota is beneficial for normal immunity’s progression, but when taking on maladjustment, it is available to motivate autoimmunity via diversified non-antigen-specific impacts on pathogenic and modulatory lymphocytes [1]. Inflammatory illnesses of the gastrointestinal tract are frequently linked with the intestinal microflora’s dysbiosis [2]. Colitis is a multi-factorial inflammatory lesion of the colon. Intestinal mucosal barrier dysfunction functions as a key part in colitis’ pathogenesis [3]. In general, the tight coupling of the mucus layer and the intestinal epithelium is the crux for maintaining the barrier’s integrity and the normal intestinal homeostasis. Meanwhile, the probable defect’s presence is available to result in intestinal inflammation of colitis [4]. Hence, repressive intestinal mucosal barrier dysfunction and motivated epithelial repair are vital for colitis cure. Nevertheless, the modulation mechanism of the intestinal mucosal barrier is obscure.

Berberine (BBR), a native compound extracted from Coptis chinensis, is available to be applied for remedying gastrointestinal illnesses like colitis [5]. Numerous studies have manifested BBR has powerful and anti-inflammatory activity, manifesting as reduced pro-inflammatory cytokines and enhancive anti-inflammatory cytokines’ concentration [6]. Additionally, BBR also has an antioxidant effect, manifesting as repressive oxidative stress [7]. In colitis, inflammation and oxidative stress are the two vital elements. Hence, BBR is appropriate for colitis cure. Though BBR’s efficacy on colitis has been verified in plentiful ways, for instance, BBR represses macrophage M1 polarization via the AKT1/SOCS1/NF-κB pathway for prevention of dextran sodium sulfate (DSS) -stimulated colitis [8]. Nevertheless, BBR’s molecular mechanism in colitis has not been completely illustrated.

MicroRNA (miRNA), a cluster of brief endogenous non-coding RNA, controls gene expression at the post-transcriptional level via combining with mRNAs’ 3’-untranslated region (UTR) [9]. Numerous studies have elucidated miRNA maladjustment is implicated in diversified human illnesses, covering colitis [10]. For instance, miR-142-5p is elevated within colitis’ progression, and silencing one is available to perfect the disease in experimental colitis mouse models [11]. MiR-223 is enhancive in experimental colitis stimulated via DSS and mitigates intestinal inflammation via targeting the IL-6/STAT3 pathway [12]. MiR-103a-3p, a type of miRNA being paid much attention, has been verified to be disparately manifested and take on a momentous character in diversified illnesses, like cerebral ischemia reperfusion injury [13], osteoarthritis [14] and cancer [15]. Nevertheless, its expression and functions in colitis are not yet distinct.

The research was to figure out BBR’s characters in colitis and its molecular mechanism. Hypothesis was proposed based on the results of *in vitro* and *in vivo* experiments: Berberine represses Wnt/β-catenin pathway activation via modulating the miR-103a-3p/Bromodomain-containing protein 4 (BRD4) axis, thereby refraining pyroptosis and reducing the intestinal mucosal barrier defect induced via colitis. The research’s results offer brand-new insights for colitis cure.

## 2. Materials and methods

### 2.1. Animals

Offer of specific pathogen-free (SPF) grade male C57BL/6 J mice (7–8 weeks old) purchased was via Hubei Provincial Center for Disease Control and Prevention. Placing the animals was with free access to food and water. All animal experiments in the research were carried out on the grounds of the guidelines of the Committee of the Institute of Zoology of Guizhou Medical University, and approval was via the Committee of Animal Protection and Use of Guizhou Medical University (Approval number: Gz-201,603,112 H).

### 2.2. DSS-stimulated mouse colitis model and grouping

**Figure 1.**
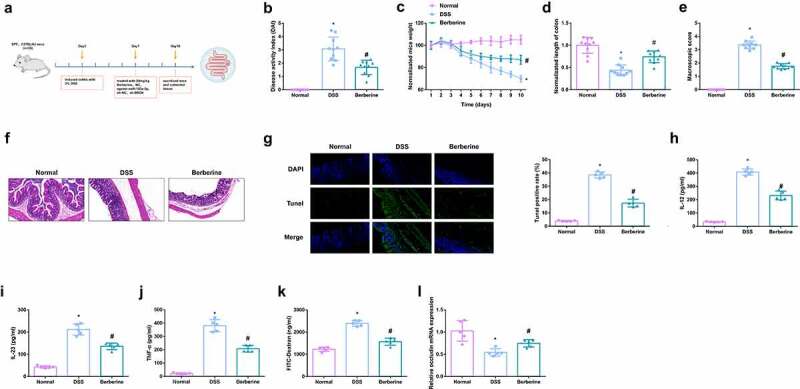
BBR relieves colitis-induced pyroptosis and intestinal mucosal barrier defects. Animal experiment flow chart. (b) DAI scores of mice in each group. (c) Normalization of mouse body weight. (d) The length of the colon of each group of mice. (e) Macroscopic score of colon tissue in each group. (f) HE staining to detect the pathological condition of colon tissue. (g) TUNEL staining to detect apoptosis. (h-j) ELISA method to detect IL-12, IL-23 and TNF-α in the serum of mice. (k) Assessment of intestinal permeability. L. qRT-PCR detection of occludin mRNA. B-E, n = 10, F-L, n = 5; The data in the Fig. were all measurement data, and manifestation of which was as mean ± SD. * vs the Normal, *P* < 0.05; # vs the DSS, *P* < 0.05.

As manifested in Figure 1a, after 3-d adaptive feeding in the center, casual assignation of C57BL/6 J mice was into 7 groups: (1) the normal control (Normal); (2) the DSS (DSS); (3) the DSS + BBR (BBR); (4) the DSS + miR-103a-3p agomir (D + miR-103a-3p); (5) the DSS + miR- 103a-3p agomir negative control (D + NC), (6) the DSS + BBR + miR-103a-3p antagomir (D + B + ant-miR-103a-3p), (7) the DSS + BBR + miR-103a-3p antagomir NC (D + B + ant-NC) (all n = 8). Colitis induction was in all mouse groups except the Normal via addition of 3.0% DSS (36–50kDa; MP Biomedicals) to drinking water for 7 d. Treatment of the mice in the BBR was with BBR hydrochloride solution (suspension, dose of 20 mg/kg) by gavage 3 d daily before tissue harvesting [16], and gavage in the mice in the model was with clear water for control. Additionally, intraperitoneal injection of mice in the miR-103a-3p and NC was with 100 μL agomir-miR-103a-3p, sh-BRD4 and their separate NCs (dissolvement in 2 mg/mL normal saline). Recording of the body weight, stool consistency and stool occult blood of all groups was every day.The method of methylaminophenol sulfate was used to detect hemafecia, and the specific operations were as follows: a small amount of mouse feces was applied to the center of the slide, and 3 drops of 10 g/L methylaminophenol sulfate solution and 3 drops of 3% hydrogen peroxide solution were dripped, and they were mixed evenly, and then the results were observed. The observation of stool consistency could be divided into three types, covering the normal, the loose and loose stools. The observation results of fecal occult blood and stool consistency were assessed using the Disease Activity Index (DAI) score, DAI = (percentage of weight loss + fecal traits score + fecal occult blood score) /3, and specific scoring rules were seen in Table 1. On the 10^th^ d, anesthetization of the mice was with ether; collection of blood was from the animal’s eyeballs, and then euthanasia of the mice was via cervical dislocation, and excision of the colon was prepared for histological analysis and other measurements [17].

**Table 1.** DAI scoring criteria

Score	Percentage of weight loss	Fecal traits	Hemafecia condition
0	0	normal	normal
1	1–5	-	-
2	6–10	loose	positive occult blood
3	11–15	-	-
4	>15	loose stools	bloody stools to the naked eyes

Stool traits: normal: granular shaped stool; loose: a semi-formed or non-anal paste of stool; loose stool: watery, and can adhere to the anus.

### 2.3. *Permeability of intestinal epithelium* in vivo

Application of Fluorescein isothiocyanate (FITC)-dextran tracer (4kDa, Sigma-Aldrich, St. Louis, MO, USA) was for assessment of intestinal permeability. After fasting, the mice were given FITC-dextran (FD4) at a dose of 20 mg/kg and incubation was before euthanasia. At the time of euthanasia, collection of blood samples and allowing to coagulate were conducted. Then, centrifugation of the sample was at 3000 rpm. Measurement of the concentration of FITC-dextran was via fluorescence spectroscopy at an excitation wavelength of 480 nm and an emission wavelength of 520 nm [18].

### 2.4. Hematoxylin-eosin (HE) staining

After fixation with 4% paraformaldehyde, dehydration, embedding, and sectioning of the mouse colon tissue were implemented. After HE staining, observation of the pathological structure of the colon was with a microscope (BA210T, Motic, Kowloon, Hong Kong) [19].

### 2.5. TdT-mediated dUTP-biotin nick end-labeling (TUNEL)

In the light of the instructions, the TUNEL cell apoptosis detection kit (Shanghai Institute of Biotechnology, Shanghai) was applied for assessment of cell apoptosis in colon tissues. In short, dewaxing of colon tissue in xylene, dehydration with graded ethanol, and then incubation with 100 mg/mL proteinase K in a wet box were put into effect. Incubation of these sections was with the TUNEL reaction mixture and observation was via transcriptional activator protein together with diaminobenzidine. The apoptosis rate is the ratio of the number of apoptotic cells per unit area to the total number of cells [20].

### 2.6. Cell culture

Obtaining of Caco-2 cells and human NCM460 cells was from INCELL and maintaining was with Roswell Park Memorial Institute 1640 covering 10% fetal bovine serum (FBS). Culture of the cells was with replacement of the medium with fresh medium every other day. Application of cells with 90% confluence was in all experiments. Application of IL-6 (10 ng/mL) *in vitro* experiments was for stimulation of Caco-2 cells and NCM460 cells and incubation was with BBR (50 mM) for treatment for 24 h [21].

### 2.7. Cell transfection

Cell transfection was with 100 nM miR-103a-3p inhibitor, elevation BRD4 vector or their NCs (all synthesis and purchase from GeneCopoeia, Guangzhou, China).Small interfering RNA (siRNA) was synthesized by Gene Pharma (Shanghai, China). Si-β-catenin was transfected into Caco-2 cells for β-catenin knockdown. NC siRNA was used as negative control. Lipofectamine 2000 (Invitrogen) was applied in the light of the manufacturer’s protocol.

### 2.8. Cell proliferation test

Seeding of cells was in 96-well plates with a concentration of 1 × 10^4^ cells/mL. Application of the cell counting kit-8 (CCK-8) kit (Dojindo Laboratories, Kumamoto, Japan) was for recording of the optical density (OD) at 450 nm at designated time points (24, 48, 72 h) to depict the viability curve [22].

### 2.9. Apoptosis detection

Seeding of the cells was in a 6-well plate with culture, and then reaction was with FITC-Annexin V and 250 μg/mL propidium iodide Pyridine (PI). Test of the cells was via flow cytometry (FACSCalibur; BD Biosciences, Franklin Lakes, NJ, USA) [23].

### 2.10. Enzyme-linked immunosorbent assay (ELISA)

Collection of Caco-2 cells and NCM460 cells was for culture of the supernatant, and harvest of blood was from mouse eyeballs. After centrifugation, detection of inflammatory factors (IL-12, IL-23, TNF-α) in the cell supernatant and mouse serum was with an ELISA kit (Abcam) [24].

### 2.11. Barrier detection

Transepithelial electrical resistance (TEER) was for measurement of the barrier’s integrity. Seeding of Caco-2 cells was in a 24-well Transwell plate on a polyester membrane filter (pore size 0.4 μM, surface area 1.12 cm^2^). Addition of the complete medium was to the root apical compartment and the basal compartment, with replacement of the complete medium every other day until the 21^st^ d. After forming a complete monolayer, the epithelial Millicell ERS-2 Volt-ohmmeter (Millipore; Bedford, MA, USA) was employed for measurement of TEER. Recording the resistance value was until obtaining three similar measurements in succession, and calculation of TEER was in ohm cm^2^ after subtracting the blank value of the membrane insert. Standardization of the TEER value is to the initial value with manifestation as a percentage of the initial resistance value [25].

### 2.12. Reverse transcription quantitative polymerase chain reaction (RT-qPCR)

Extraction of total RNA from tissues and cells was via TRIzol reagent (Thermo Fisher Scientific, Inc.). Reverse transcription of RNA was via RevertAid First Strand complementary DNA (cDNA) Synthesis Kit (K1622, Thermo Scientific). RT-qPCR was via SYBR Green PCR Master Mix (Applied Biosystems, USA) and BioRad CFX-96 real-time PCR system (BioRad, USA). Primers were manifested in Table 2. Normalization of miRNA and mRNA was to U6 and glyceraldehyde-3-phosphate dehydrogenase (GAPDH), separately. Calculation of the relative RNA was via 2^−ΔΔCT^ method [26].

**Table 2.** RT-qPCR primer sequence

Genes		Forward (5’-3’)	Reverse (5’-3’)
BRD4	Mice	GGACGAGGGAGGAAAGAAACA	AGGAAAGGGGTGAGTTGTGG
	Human	GTGGTGCACATCATCCAGTC	CCGACTCTGAGGACGAGAAG
GAPDH	Mice	TGGCCTTCCGTGTTCCTAC	GAGTTGCTGTTGAAGTC
	Human	TGTGGGCATCAATGGATTTGG	ACACCATGTATTCCGGGTCAAT
MiR-103a-3p	Mice	AGCAGCATTGTACAGGGCTATGAA	TGGTGTCGTGGAGTCG
	Human	AGCAGCATTGTACAGGGCTA TGAA	TGGTGTCGTGGAGTCG
U6	Mice	TCGCACAGACTTGTGGGAGAA	CGCACATTAAGCCTCTATAGTTACTAGG
	Human	CTCGCTTCGGCAGCACA	AACGCTTCACGAATTTGCGT

### 2.13. Western blot

Application of Radio-Immunoprecipitation assay lysis buffer and protease inhibitor mixture (Roche, Basel, Switzerland) was for extraction of total protein from colon tissues and cells. Then measurement of the protein concentration was via the bicinchoninic acid protein determination kit (ASPEN, Wuhan, China) in the light of the manufacturer’s instructions. Separation of the protein was via 10% sodium dodecyl sulfate polyacrylamide gel electrophoresis. Subsequently, electro-blot of the protein was onto a polyvinylidene fluoride membrane blocked with 5% skim milk, and then application was with appropriate anti-BRD4 (ab128874), β-catenin (ab32572), GAPDH (ab8245; Abcam) (all 1: 1000). Incubation of the peroxidase-conjugated secondary antibody was with the membrane. Finally, visualization and analysis of the target protein bands were conducted [27].

### 2.14. The luciferase activity assay

Synthesis of the wild-type (WT) 3’ UTR of BRD4 cDNA was via polymerase chain reaction (PCR) and clone was into pmmir-report luciferase for generation of WT-BRD4 3ʹUTR. Production of BRD4 3’ was in the light of a mutant of WT-BRD4 3ʹUTR and name of the resulting vector was MUT-BRD4 3ʹUTR. Transient transfection of Caco-2 cells was with Tese vector (pmmir-report plasmid, WT/MUT-BRD4 3ʹUTR) and miR-103a-3p or miR-NC applying Lipofectamine 3000 reagent (Invitrogen, Carlsbad, CA, USA). Then Promega (Madison, WI, USA) was employed for detection of luciferase activity [28].

### 2.15. Statistical analysis

Application of SPSS 21.0 (SPSS, Inc, Chicago, IL, USA) statistical software was for analysis of the data. After Kolmogorov-Smirnov test, the data were normally distributed, and manifestation of the results was as mean ± standard deviation (SD). Two-group comparison was via t test, while the comparison among multiple groups was via one-way analysis of variance (ANOVA), and Fisher’s least significant difference t test (LSD-t) was applied for the pairwise comparison after ANOVA analysis. Manifestation of enumeration data was via rate or percentage, and the chi-square test was employed for comparative analysis. *P* was two-sided test; *P* < 0.05 emphasized obvious statistical meaning.

## 3. Results

Here, it was aimed to investigate the functional role of BBR in colitis and the regulatory mechanism by which BBR repressed the activation of Wnt/β-catenin signaling pathway by regulating miR-103a-3p/BRD4 axis. A series of *in vitro* and *in vivo* experiments were conducted, and it was found that Berberine repressed pyroptosis and alleviated intestinal mucosal barrier defects caused by colitis, by inhibiting activation of Wnt/β-catenin signaling pathway by regulating miR-103a-3p/BRD4 axis. Therefore, in the data it is the first to investigate the function and mechanism of BBR in colitis via miR-103a-3p/BRD4 axis, providing new insights into the pathogenesis of colitis.

### 3.1. BBR relieves colitis-induced pyroptosis and intestinal mucosal barrier defects

In the research, the colitis model induction was via DSS at the beginning of the experiment and BBR was given.Prior to this, BBR safety was evaluated and mice were given BBR orally in a single dose (suspension, 20 mg/kg). After close monitoring, no animals died within 7 days after treatment. Body weight was not significantly affected, and the animals’ fur was shiny and the behavior of the mice was not affected. Recording of the body weight, stool consistency, stool occult blood, and general physical symptoms of mice in the all groups was every day. The flow chart of animal experiments was shown in Figure 1a. First, the observation results of fecal occult blood and stool consistency were evaluated by disease activity index (DAI) score. The results showed that DAI score of mice with DSS-induced colitis was significantly increased, and certain remission was achieved after BBR treatment (Figure 1b). As clarified in Figure 1c, vs. normal mice, lost weight was presented in mice of the DSS. Nevertheless, after the administration of BBR, the weight loss was gradually reduced from the 7^th^ d. Vs. the normal, the length of the colon in the DSS was declined, hinting that DSS mice had acute colonic inflammation, while the average colon length in the BBR was 1.21 times that of the DSS (Figure 1d). In the BBR, the gross damage score, and the colonic mucosal damage were reduced, which was 76% of that in the DSS (Figure 1e). It came out DSS treatment stimulated colon damage, while the BBR had less colonic inflammatory cell infiltration and apoptosis, and good structural integrity (Figures. 1f,g). Pyroptosis is a familiar programmed cell death mechanism of congenital immune response, manifesting as the morphological features of apoptosis and inflammatory necrosis [29]. Detection of inflammatory factors in the serum of mice clarified apparent elevation, and inflammation was clearly repressed after BBR treatment (Figures 1h-j). For evaluation of colitis-stimulated intestinal mucosal barrier defect, detection of the intestinal permeability was conducted, and it turned out BBR declined the flux of FD4 in the blood of colon mice (Figure 1k). Additionally, studies have illustrated the tight junction (TJ) protein occludin is available to help maintain the intestinal barrier function [30]. Occludin was memorably repressed in the DSS but elevated in the BBR (Figure 1l). The above results elaborated BBR relieved colitis-induced pyroptosis and intestinal mucosal barrier defects.

### 3.2. MiR-103a-3p eases DSS-stimulated colitis

**Figure 2.**
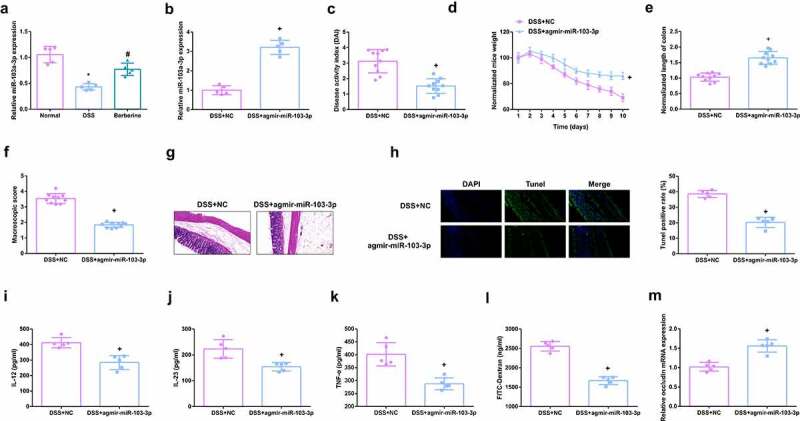
MiR-103a-3p eases DSS-stimulated colitis. A/B. qPCR to detect miR-103a-3p in mouse colon tissue. C. DAI scores of mice in each group. D. Normalization of mouse body weight. E. The length of the colon of each group of mice. F. Macroscopic score of colon tissue in each group. G. HE staining to detect the pathological condition of colon tissue. H. TUNEL staining to detect apoptosis. I-K. ELISA method to detect IL-12, IL-23 and TNF-α in the serum of mice. L. Assessment of intestinal permeability. M. qRT-PCR detection of occludin mRNA. C-F, n = 10; A&B&G-M, n = 5; The data in the Fig. were all measurement data, and manifestation of which was as mean ± SD. + vs the DSS+NC, *P* < 0.05.

Detection of miR-103a-3p in DSS-induced mouse colon tissue, manifested the reduction, and apparent elevation after BBR gavage (Figure 2a). For sake of further studying miR-103a-3p’s impacts on mouse colitis, injection of miR-103a-3p agmir and its NC was into DSS mice, with verification (Figure 2b). It was found that DAI score in mice with colitis was significantly decreased after up-regulation of miR-103a-3p (Figure 2c). The experiments discovered miR-103a-3p alleviated the weight loss of mice (Figure 2d); The length of the colon was longer vs. the DSS mice (Figure 2e), the gross damage score (Figure 2f), the infiltration of colonic inflammatory cells and apoptosis were declined (Figures. 2g,h). Moreover, inflammatory factors in the serum of mice were memorably reduced (Figures 2i-k), and the intestinal mucosal barrier was mitigated (Figures. 2l,m). In brief, miR-103a-3p eases DSS-stimulated colitis.

### 3.3. MiR-103a-3p targets BRD4

**Figure 3.**
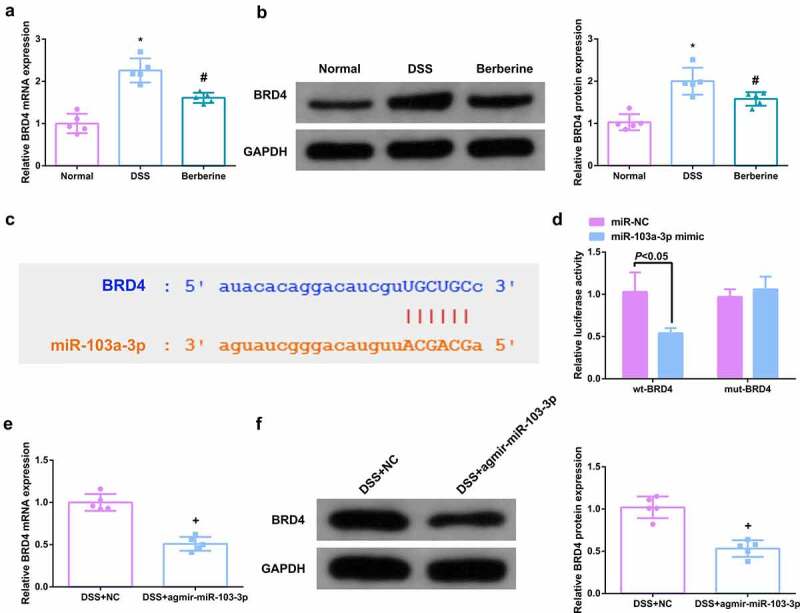
MiR-103a-3p targets BRD4. A/B. qPCR to detect BRD4 in mouse colon tissue. B. Western Blot detection of BRD4 in mouse colon tissue. C. Bioinformatics website forecast of the binding site of miR-103a-3p with BRD4. D. The luciferase activity assay verification of the targeting link of miR-103a-3p with BRD4, N = 3. E/F. qPCR and Western Blot detection of BRD4 in mouse colon tissue with elevated miR-103a-3p. n = 5; The data in the Fig. were all measurement data, and manifestation of which was as mean ± SD. * vs the Normal, *P* < 0.05. # vs. the DSS, *P* < 0.05. + vs the DSS+NC, *P* < 0.05.

Elevated BRD4 was tested in DSS-induced colitis mice, which is the opposite of miR-103a-3p (Figures 3a, b). Considering the link of miR-103a-3p and BRD4, TargetScan software was employed to figure out miR-103a-3p’s targets. As manifested in Figure 3c, it was determined the latent binding sites of miR-103a-3p in the 3ʹUTR of BRD4. For further verification, detection of whether miR-103a-3p immediately targeted the 3ʹUTR of BRD4 was conducted. The results clarified after co-transfection of 3ʹUTR luciferase reporter gene and miR-103a-3p mimic in Caco-2 cells, the activity of luciferase reporter gene was clearly reduced, but when the 3ʹUTR of BRD4 was mutated, no difference was presented (Figure 3d). Moreover, a reduction in BRD4 was discovered in the colon tissue of colitis mice elevating miR-103a-3p (Figures 3e, f), elaborating that miR-103a-3p targeted BRD4.

### 3.4. Depressive BRD4 mitigates DSS-induced colitis damage

**Figure 4.**
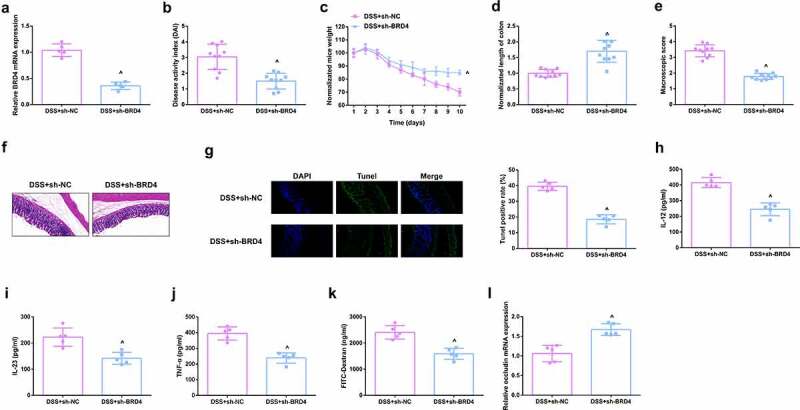
Depressive BRD4 mitigates DSS-induced colitis damage. A.qPCR detection of BRD4 after repressing BRD4. B. DAI scores of mice in each group. C.Normalization of mouse body weight. D. The length of the colon of each group of mice. E. Macroscopic score of colon tissue in each group. F. HE staining to detect the pathological condition of colon tissue. G. TUNEL staining to detect apoptosis. H-J. ELISA method to detect IL-12, IL-23 and TNF-α in the serum of mice. K. Assessment of intestinal permeability. L. qRT-PCR detection of occludin. B-E, n = 10; A&B&F-L, n = 5; The data in the Fig. were all measurement data, and manifestation of which was as mean ± SD. + vs the DSS+sh-NC, *P* < 0.05.

For studying BRD4ʹs impacts on mouse colitis, repressive BRD4 was in mice (Figure 4a), and it came out the reduction of BRD4 reduced DAI scores (Figure 4b), alleviated the weight loss of mice (Figure 4c), and the length of the colon was longer vs. DSS mice (Figure 4d), reduced gross damage score (Figure 4e), colonic inflammatory cell infiltration, and apoptotic cells were presented (Figures 4f-g). Moreover, inflammatory factors in the serum of mice were memorably reduced (Figures 4h-j), and the intestinal mucosal barrier was mitigated (Figure. 4k,l). The above results clarified repressive BRD4 mitigated DSS-induced colitis damage.

### 3.5. *BBR motivates cell advancement, and alleviates intestinal barrier damage in IL-6-induced colitis model* in vitro

**Figure 5.**
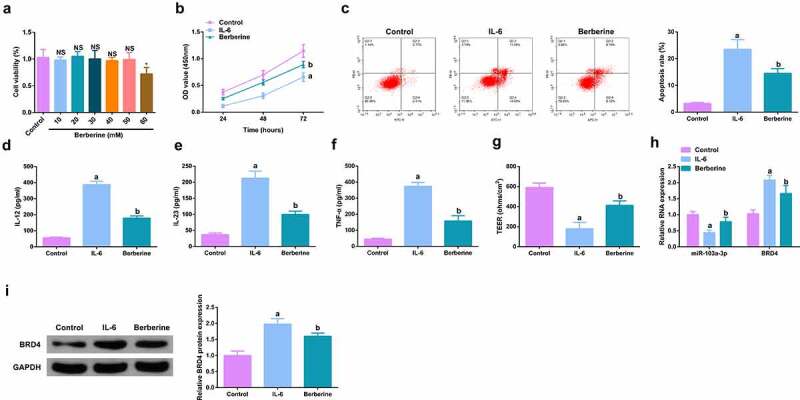
BBR motivates cell advancement, and alleviates intestinal barrier damage in IL-6-induced colitis model *in vitro*. A. CCK-8 method to determine the cytotoxicity of BBR (0–60 mM) to NCM460 cells. B. CCK-8 method to determine the cell viability of each group. B. Measurement of apoptosis via flow cytometry. C-E. ELISA detection of inflammatory factors in the cell supernatant. F. TEER measurement of the barrier’s integrity. G/H. qPCR and Western Blot detection of miR-103a-3p with BRD4 in cells of each group. N = 3; The data in the Fig. were all measurement data, and manifestation of which was as mean ± SD; a vs the Control, *P* < 0.05; b vs. the IL-6, *P* < 0.05.

For studying the therapeutic action of BBR on colitis *in vitro*, IL-6 (10 ng/ml) was applied for stimulating Caco-2 cells and NCM460 cells to obtain an *in vitro* model, and incubation of the cells was with BBR (50 mM). Prior to this, it was first evaluated the toxicity of BBR. CCK-8 was applied to detect the cytotoxicity of BBR (0–60 μM) to Caco-2 and NCM460 cells and it was found no toxicity at BBR concentrations less than 50 mM (Figure 5a, Attached Figure 1a). It turned out the cell advancement of the IL-6 group was memorably restrained, but strengthened after BBR treatment (Figures 5b,c, Attached Figures 1b,c). Meanwhile, inflammation in the IL-6 group was clearly elevated, but apparently restrained after BBR treatment (Figures 5d-f, Attached Figures 1d-f). Simultaneously, BBR could also reverse the decline in TER stimulated via IL-6 (Figure 5g, Attached Figure 1g). Moreover, BBR treatment reduced IL-6-induced repressive miR-103a-3p and elevated BRD4 (Figures 5h-j, Attached Figures 1h-j). In short, BBR motivated cell advancement, and alleviated intestinal barrier damage in IL-6-induced colitis model *in vitro*.

### 3.6. Repressive miR-103a-3p or enhancive BRD4 turned around BBR’s therapeutic action

**Figure 6.**
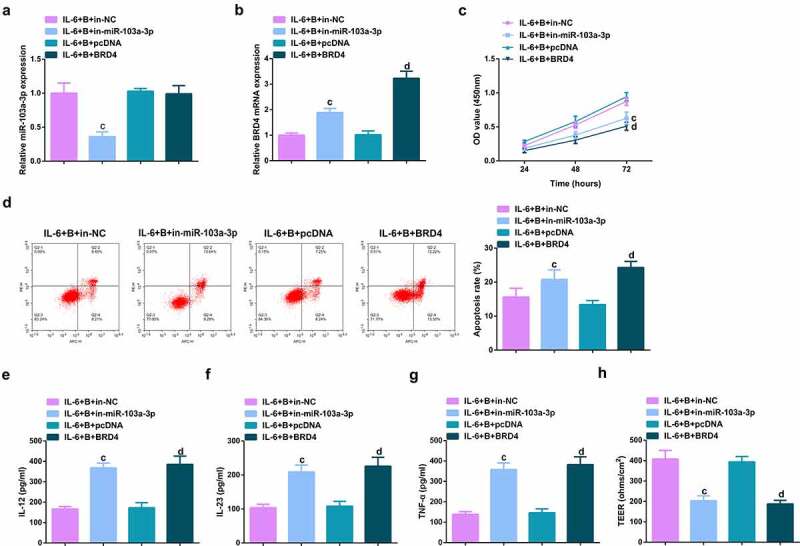
Depressive miR-103a-3p or enhancive BRD4 turned around BBR’s therapeutic action. A/B. qPCR to verify transfection efficiency. C. CCK-8 method to determine the cell viability of each group. D. Measurement of apoptosis via flow cytometry. E-G. ELISA detection of inflammatory factors in the cell supernatant. H. TEER measurement of the barrier’s integrity. N = 3; The data in the Fig. were all measurement data, and manifestation of which was as mean ± SD. c vs the IL-6 + B+ in-NC, *P* < 0.05. d vs. the IL-6 + B+ pcDNA, *P* < 0.05.

**Figure 7.**
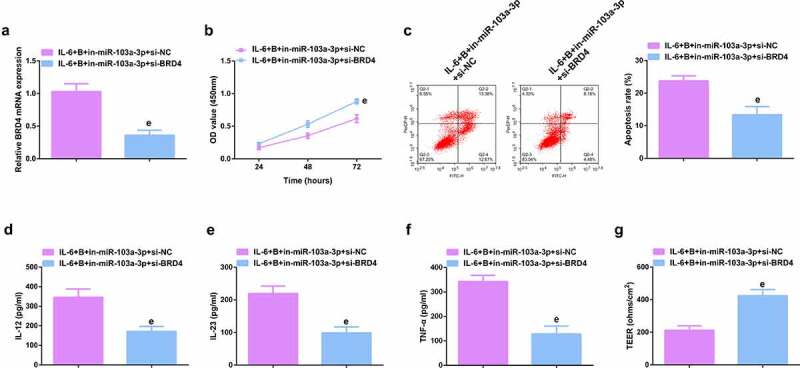
Depressive miR-103a-3p or enhancive BRD4 turns around BBR’s therapeutic action. A. qPCR to verify transfection efficiency. B. CCK-8 method to determine the cell viability of each group. C. Measurement of apoptosis via flow cytometry. D-F. ELISA detection of inflammatory factors in the cell supernatant. G. TEER measurement of the barrier’s integrity. N = 3; The data in the Fig. were all measurement data, and manifestation of which was as mean ± SD. c vs. the IL-6 + B+ in-NC, *P* < 0.05. e vs. the IL-6 + B+ in-miR-103a-3p+si-NC, *P* < 0.05.

To further figure out BBR’s molecular mechanism in colitis cure, transfection of the miR-103a-3p inhibitor and BRD4 elevation vector and their separate NC was into Caco-2 cells stimulated via IL-6, and co-culture was with BBR with verification of the transfection efficiency (Figures 6a,b, Attached Figures. 2a,b). Experiments have illustrated repressive miR-103a-3p or elevated BRD4 restrained the cell advancement of BBR treatment with elevated inflammation, and aggravated the damage of the intestinal barrier, and apparently turned around BBR treatment’s efficacy on IL-6 stimulated Caco-2 cells (Figures 6c-h, Attached Figures 2c-h).In addition, BRD4 was also inhibited in cells with repressive miR-103a-3p to study the effect of miR-103a-3p/BRD4 deletion on BBH, and the decrease of BRD4 mRNA was detected by qPCR (Figure 7a, Attached Figure 3a). The experiment found that, inhibition of BRD4 reversed the effect of depressive miR-103a-3p on BBR, resulting in increased cell proliferation (Figure 7b, Attached Figure 3b), decreased apoptosis (Figure 7c, Attached Figure 3c), repressive inflammatory factors (Figures 7d-f, Attached Figures 3d-f), and reduced intestinal barrier damage (Figure 7g, Attached Figure 3g).

### 3.7. BBR restrains Wnt/β-catenin pathway via modulating the miR-103a-3p/BRD4 axis

**Figure 8.**
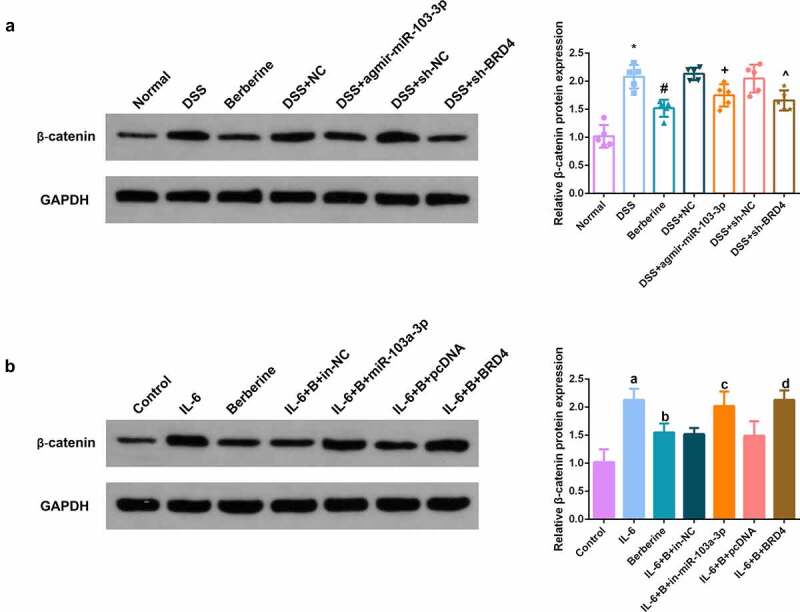
BBR restrains Wnt/β-catenin pathway via modulating the miR-103a-3p/BRD4 axis. A, B. Western Blot detection of β-catenin in colon tissues of each group, *in vivo* experiment, n = 5; *in vitro* experiment, N = 3. The data in the Fig. were all measurement data, and manifestation of which was as mean ± SD. * vs. the Normal, *P* < 0.05. # vs. the DSS, *P* < 0.05. + vs. the D+ NC, *P* < 0.05; a vs. the control, *P* < 0.05. b vs. the IL-6, *P* < 0.05; c vs. the IL-6 + B+ in-NC, *P* < 0.05. d vs. the IL-6 + B+ pcDNA, *P* < 0.05.

In the dynamic balance of intestinal epithelial cell advancement, the Wnt/β-catenin pathway is deemed to play a crucial control role [31]. Detection of Wnt/β-catenin pathway *in vivo* and *in vitro* models manifested BBR, elevated miR-103a-3p or constraining BRD4 was available to clearly repress Wnt/β-catenin pathway; Depressive miR-103a- 3p or enhancive BRD4 turned around BBR’s repression on Wnt/β-catenin (Figure 8).

### 3.8. *Knockdown β-catenin further promotes the protective effect of Berberine on colitis* in vitro

**Figure 9.**
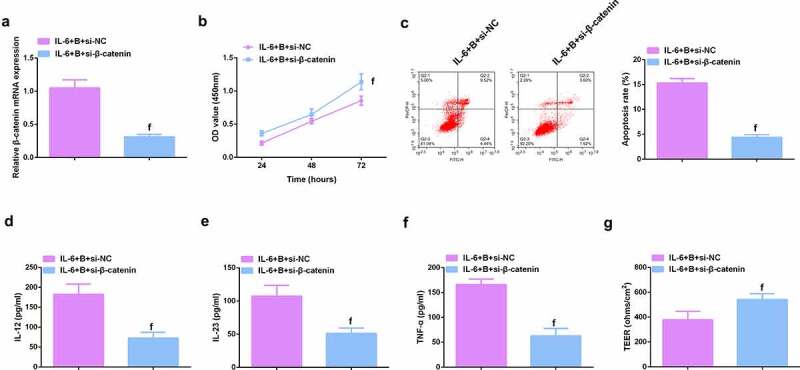
β-catenin knockdown further promotes the protective effect of Berberine on colitis *in vitro*. **A**. qPCR detection of β-catenin mRNA expression after knocking down β-catenin; B. CCK-8 to detect the proliferation ability of cells after knocking down β-catenin; C. Flow cytometry to detect cell apoptosis after knocking down β-catenin; D-F. ELISA to detect the levels of inflammatory cytokines (IL-12, IL-23, TNF-α) in the supernatant of cells after β-catenin knockdown; G. The level of inflammatory cytokines (IL-12, IL-23, TNF-α) in cell supernatant fluid, N = 3. The data in the Fig. were all measurement data, and manifestation of which was as mean ± SD; f, vs. the IL-6 + B = si-NC group, *P* < 0.05.

To further study the role of Wnt/β-catenin signaling pathway in colitis, it was used siRNA to knock down β-catenin in IL-6-induced Caco-2 and NCM460 cell models, and a significant decrease in β-catenin mRNA was detected by qPCR (Figure 9a, Attached Figure 4a). It was found that si-β-catenin further enhanced the role of Berberine in colitis, further increased cell proliferation ability (Figure 9b, Attached Figure 4b), and apoptosis was further reduced (Figure 9c, Attached Figure 4c), and inflammatory cytokines (IL-12, IL-23 and TNF-α) levels in cell supernatant fluid were further decreased (Figures 9d-f, Attached Figures 4d-f), and TER level was further increased (Figure 9g, attached Figure 4g). These experiments suggested that knocking down β-catenin further promoted the protective effect of Berberine on colitis *in vitro*.

## 4. Discussion

BBR’s characters in colitis have been extensively verified, covering its ability to mitigate the intestinal barrier function [32], elevate anti-inflammatory and anti-oxidative stress responses [33], modulate dysfunctional bacteria and functions [34], etc. In the research, it was discovered that administration of BBR reduced the colonic mucosal damage in mice with DSS-induced colitis, inflammatory cell infiltration, pyroptosis, and inflammation. Numerous studies have manifested drugs are available to treat diseases via miRNAs. Ming Sui *et al*. [35] report BBR alleviates hepatic insulin resistance via modulating the miR-146b/SIRT1 pathway. Yan Xia *et al*. [36] announce BBR refrains bladder cancer cell advancement via elevating miR-17-5p and restraining JAK1-STAT3 signaling. Here, it was discovered that BBR treatment elevated miR-103a-3p, and further testified that miR-103a-3p targeted BRD4. These results elaborated BBR efficaciously relieved colitis-induced pyroptosis and intestinal mucosal barrier defects via targeting modulation of miR-103a-3p/BRD4 axis. As far as we know, this is the first study to discover that BBR impacts colitis’ progression via modulating miRNA.

First, induction of a mouse model of colitis was via DSS. DSS is a polyanionic derivative, which is linked with macrophage dysfunction and intestinal flora imbalance. Its toxic effect on colonic epithelium stimulates cell erosion and apoptosis, resulting in reduced colonic barrier function [37]. It was discovered that DSS-treated mice developed acute colonic inflammation, weight loss, colon length reduction, obviously damaged colonic mucosa, and enhancive colonic inflammatory cell infiltration. Pyroptosis, a type of inflammatory programmed cell death, is mediated via diversified inflammasomes, which is available to discern danger signals and activate the secretion of pro-inflammatory cytokines [38]. Its major function is to stimulate a strong inflammatory response and guard the host from microbial infections, but undue pyroptosis can cause various inflammatory illnesses [39]. Pyroptosis is also manifested as the morphological features of apoptosis and inflammatory necrosis [29]. In experiments, it was discovered repressive cell advancement in colon tissue of DSS mice, with elevated inflammation in mouse serum. Intestinal mucosal barrier dysfunction is the vital pathology of colitis. The intestinal epithelial lining composed of tightly continuous epithelial cell layers performs as a physical barrier. Once disordered, it will lead to further inflammation and intemperate secretion of chemokines and adhesion molecules [40]. The permeability of FD4 is a crucial indicator for evaluating intestinal permeability [41]. Occludin is a momentous part of TJ proteins and is sort out as a transmembrane protein immediately paricipating in paracellular transport, which can maintain the intestinal mucosal barrier function [42]. It was detected that the flux of FD4 in the blood of DSS-treated mice was elevated, but occludin was reduced. While BBR turned around DSS’s impacts, manifesting as relieved colitis-induced pyroptosis and intestinal mucosal barrier defects. Meanwhile, *in vitro* experiments clarified BBR reversed IL-6ʹs impacts, elevated Caco-2 cell advancement with refrained inflammation. In short, BBR was available to restrain pyroptosis and alleviate intestinal barrier damage, thereby mitigating colitis model. Meanwhile, via further experimental verification, BBR functioned in colitis via modulating the miR-103a-3p/BRD4 axis.

Since miR-103a-3p was discovered, the focus of research has been to figure out its functions in cancer. Recently, elevated evidences have proved miR-103a-3p also has an irreplaceable function in certain inflammatory illnesses. For instance, miR-103a-3p drives angiotensin II–induced renal inflammation and fibrosis via the SNRK/NF-κB/p65 modulatory axis [43]. Additionally, miR-103a-3p is also linked with mitochondrial damage, apoptosis, and the secretion of inflammatory factors [44]. Down-regulating miR-103a-3p is available to mitigate lipopolysaccharide (LPS)-induced septic liver damage via repressing apoptosis, inflammation and oxidative stress [45]. In the research, it was discovered originally that miR-103a-3p was repressed in the colon tissue of colitis mice. Elevated miR-103a-3p was available to relieve the weight loss of mice, elevate the length of the colon, reduce inflammatory cell infiltration and motivate cell advancement with repressive inflammation, and the intestinal mucosal barrier was lightened; Refraining miR-103a-3p weakened the therapeutic action of BBR on colitis. These results suggested miR-103a-3p is supposed to be applied as a brand-new diagnostic biomarker and latent therapeutic target for colitis. Meanwhile, via further experimental verification, miR-103a-3p functioned in colitis via targeting BRD4.

BRD4 is considered a part of the bromodomain and extra-terminal family, which has the ability to hold high-order chromatin structure and modulate gene [46]. Some studies have manifested BRD4ʹs crucial functions in colitis’ progression. Li Chen *et al*. [47] discover repressive BRD4 can block LPS-induced colonic TJ barrier dysfunction, pyroptosis and inflammation. LncRNA UCA1 expedites the progression of ulcerative colitis via mediating the miR-331-3p/BRD4 axis [48]. In the research, it was discovered BRD4 was elevated in colitis mice. Knockdown BRD4 turned around DSS’s impacts, while elevated one reversed the therapeutic action of BBR treatment on IL-6 stimulated Caco-2 cells.

Wnt/β-catenin signal transduction is an overly conserved pathway, which is implicated in various biological processes in different cells [49]. Plentiful studies have manifested the accurate coordination of Wnt/β-catenin signal is the crux to hold intestinal homeostasis, and the maladjustment is supposed to result in inflammation or tumorigenesis [50]. In the ‘Wnt-off’ state, β-catenin will accumulate in the cytoplasm, while in the ‘Wnt-on’ state, β-catenin will elevate and accumulate in the nucleus [51]. Meanwhile, Wnt signal activation will enable the activation of lymphoid enhancer factor/T cytokine target genes [52]. Moparthi L *et al*. display, the interactions between Wnt ligands and cytokines have the potential to reveal novel therapeutic options for chronic colitis and other inflammatory diseases [53]. In the research, BBR, elevated miR-103a-3p or repressive BRD4 was available to clearly repress Wnt/β-catenin pathway; Refraining miR-103a-3p or elevated BRD4 turned around BBR’s repression on Wnt/β-catenin. The above results clarified BBR repressed Wnt/β-catenin pathway activation via modulating the miR-103a-3p/BRD4 axis, thereby refraining pyroptosis and reducing the intestinal mucosal barrier defect induced via colitis. Elucidating the range and mechanism of secretion of Wnt signaling next to intestinal tract has the potential to broaden the understanding of epithelial homeostasis and may be of particular relevance to diseases such as inflammatory bowel disease and colitis-associated cancers [54]. It was further studied the effect of β-catenin on colitis by knocking down β-catenin in cells. The results showed that knocking down β-catenin further promoted the protective effect of Berberine on colitis *in vitro*, further increased cell proliferation ability, further reduced apoptosis, and inflammatory factors (IL-12, IL-23, TNF-α) levels in cell supernatant fluid were further declined, and TER level was further elevated. Of course, regulation of Wnt signaling can also be conducted by pathway inhibitors, Hiremath IS *et al*. provide insights into different classes of inhibitors of the Wnt/β-catenin pathway [55]. We will have further discussions when conditions permit in the future.Nevertheless, the results still have some limitations. First, owing to limited laboratory conditions, the sample size is insufficient. No separate exploration of Wnt/β-catenin signaling’ s function in colitis and the mechanism of miR-103a-3p/BRD4 modulating Wnt/β-catenin signaling is manifested. Moreover, no further exploration of the downstream factors of Wnt/β-catenin signaling is clarified.

## 5. Conclusion

All in all, the research suggests a brand-new modulatory mechanism for BBR in colitis. That is to say, BBR represses Wnt/β-catenin pathway activation via modulating the miR-103a-3p/BRD4 axis, thereby refraining pyroptosis and reducing the intestinal mucosal barrier defect induced via colitis. The research conveys BBR’s function in colitis from a new perspective of pyroptosis, and miR-103a-3p is supposed to offer as a latent molecular biomarker for colitis and as an adjuvant therapeutic target for BBR.

**Image 1.**
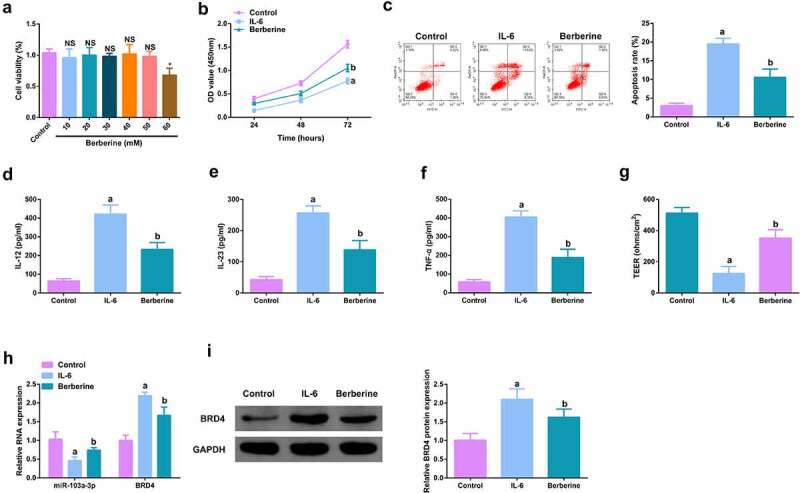
**BBR motivates cell advancement, and alleviates intestinal barrier damage in IL-6-induced colitis model *in vitro.*** A.qPCR detection of BRD4 after repressing BRD4. B. Normalization of mouse body weight. C. The length of the colon of each group of mice. D. Macroscopic score of colon tissue in each group. E. HE staining to detect the pathological condition of colon tissue. F. TUNEL staining to detect apoptosis. G-I. ELISA method to detect IL-12, IL-23 and TNF-α in the serum of mice. J. Assessment of intestinal permeability. K. Western Blot detection of occludin. C-E, n = 10; A&B&F-L, n = 5; The data in the Fig. were all measurement data, and manifestation of which was as mean ± SD. + vs. the DSS+sh-NC, *P* < 0.05.

**Image 2.**
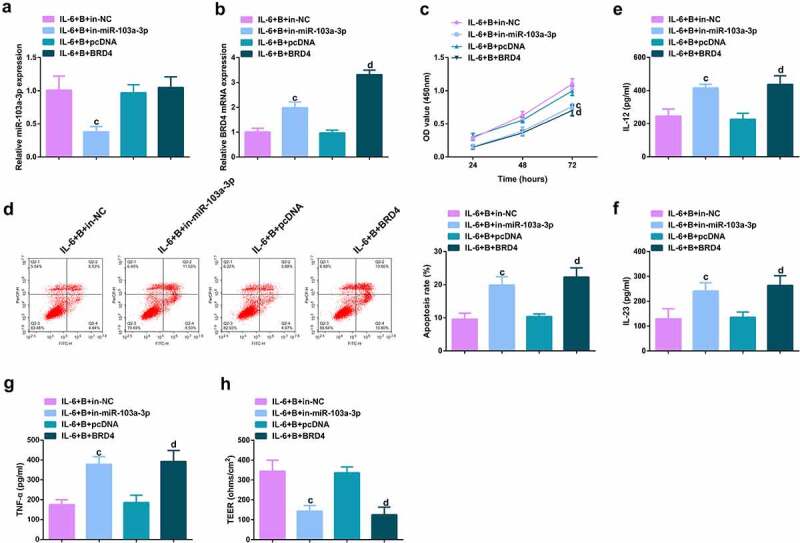
**Depressive miR-103a-3p or enhancive BRD4 turns around BBR’s therapeutic action**. A/B. qPCR to verify transfection efficiency. C. CCK-8 method to determine the cell viability of each group. D. Measurement of apoptosis via flow cytometry. E-G. ELISA detection of inflammatory factors in the cell supernatant. H. TEER measurement of the barrier’s integrity. N = 3; The data in the Fig. were all measurement data, and manifestation of which was as mean ± SD. c vs. the IL-6 + B+ in-NC, *P* < 0.05. d vs. the IL-6 + B+ pcDNA, *P* < 0.05.

**Ima7ge 3.**
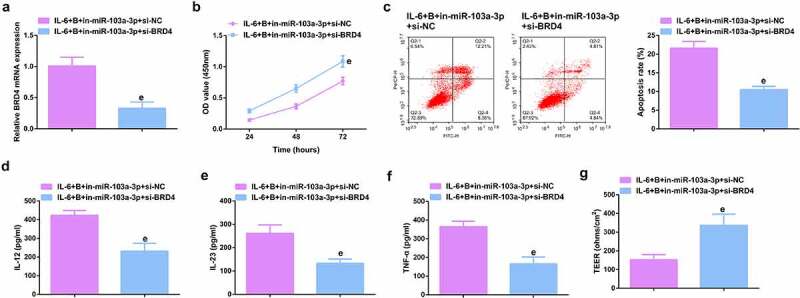
**Depressive miR-103a-3p or enhancive BRD4 turns around BBR’s therapeutic action**. A. qPCR to verify transfection efficiency. B. CCK-8 method to determine the cell viability of each group. C. Measurement of apoptosis via flow cytometry. D-F. ELISA detection of inflammatory factors in the cell supernatant. G. TEER measurement of the barrier’s integrity. N = 3; The data in the Fig. were all measurement data, and manifestation of which was as mean ± SD. c vs. the IL-6 + B+ in-NC, *P* < 0.05. e vs. the IL-6 + B+ in-miR-103a-3p+si-NC, *P* < 0.05.

**Image 4.**
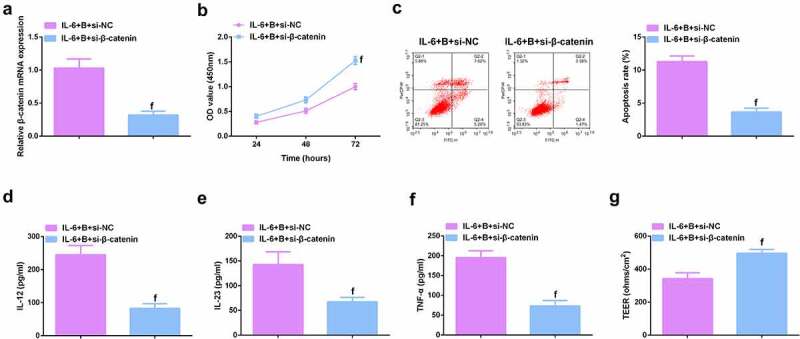
**Knockdown β-catenin further promotes the protective effect of Berberine on colitis *in vitro.*** A.qPCR detection of β-catenin mRNA expression after β-catenin knockdown; B. CCK-8 to detect the proliferation ability of cells after β-catenin knockdown; C. Flow cytometry to detect cell apoptosis after β-catenin knockdown; D-F. ELISA to detect the levels of inflammatory factors (IL-12, IL-23 and TNF-α) in the supernatant of cells after β-catenin knockdown; G. The levels of inflammatory factors (IL-12, IL-23, TNF-α) in cell supernatant fluid. N = 3; The data in the Fig. were all measurement data, and manifestation of which was as mean ± SD. f, vs. the IL-6 + B = si-NC, *P* < 0.05.
